# Immune Deviation in the Decidua During Term and Preterm Labor

**DOI:** 10.3389/fimmu.2022.877314

**Published:** 2022-06-10

**Authors:** Ying Zha, Haiyi Liu, Xingguang Lin, Long Yu, Peng Gao, Yuqi Li, Min Wu, Xun Gong, Xinyi Bian, Qi Kang, Pan Zhi, Xiaohe Dang, Jingyu Wang, Ling Feng, Fuyuan Qiao, Yafei Huang, Wanjiang Zeng

**Affiliations:** ^1^Department of Obstetrics and Gynecology, Tongji Hospital, Tongji Medical College, Huazhong University of Science and Technology, Wuhan, China; ^2^Department of Pathogen Biology, School of Basic Medicine, Tongji Medical College, Huazhong University of Science and Technology, Wuhan, China

**Keywords:** decidua, macrophage, Th cells, Treg cells, term labor, preterm labor

## Abstract

The maternal-fetal immune disorder is considered to be an important factor of preterm birth (PTB); however, the underlying mechanism is still not fully understood. This study was designed to explore the innate and adaptive immune features in the decidua during term and preterm labor. Women delivered at term or preterm were classified into four groups: term not in labor (TNL, N=19), term in labor (TL, N=17), preterm not in labor (PNL, N=10), and preterm in labor (PIL, N=10). Decidua basalis and parietalis were collected and analyzed for macrophage subtypes (M1 and M2) as well as T helper 1 (Th1), Th2, Th17 and regulatory T (Treg) cells by flow cytometry and immunohistochemistry. Our results demonstrated significantly decreased frequencies of M2 cells and elevated M1/M2 ratio in the PIL group compared to that in the PNL group in both decidua basalis and parietalis, whereas no significant differences were found between the above two groups in both sites in terms of the polarization status of Th cells. On the contrary, macrophage subsets were comparable in the TL and TNL groups, whereas elevated Th1 percentages and Th1/Th2 ratio were observed in TL women compared to that in TNL women in the decidua. Interestingly, although the frequencies and ratios of Th17 and Treg were comparable among the four groups, the Th17/Treg ratios of these groups were significantly increased in decidua basalis than that in decidua parietalis. Collectively, the M1/M2 imbalance is associated with the breakdown of maternal-fetal immune tolerance during PTB, whereas the aberrant Th1/Th2 profile plays an important role in immune disorder during term labor. Moreover, Th17/Treg deviation is more remarkable in decidua basalis than in decidua parietalis.

## Introduction

The maternal and fetal health is one of the cornerstones for the healthy development of the next generation and the sustainable development of society (1). Although remarkable achievements have been made in reducing neonatal mortality in the past 20 years, people are still confronted with the challenge of neonatal health problems such as preterm birth (PTB), which has become an increasingly important issue (2). The rate of PTB ranges from 5% to 18% across 184 countries, with an estimated 15 million babies born preterm every year and the number is still rising (3). Consequently, PTB complications (e.g., lung dysplasia, infection, brain damage, nutritional and metabolic disorders) were reported to be the leading cause of under-5 mortality (4), and the lifelong disabilities caused by PTB may bring a poor quality of life and a heavy burden on both families and society. Therefore, in order to improve neonatal and child health, there is an urgent need to reduce the PTB rate worldwide, which is dependent on the comprehensive understanding of the underlying mechanism of the pathogenesis of PTB.

Till now, spontaneous PTB has been reported to be attributable to multiple factors that influence the parturition process including infection, uterine overdistension, and the breakdown of maternal-fetal tolerance, of which the latter is considered as the driving force of PTB (5–7). During pregnancy, placentation results in the intimate contact between maternal and fetal cells at the maternal-fetal interface, which also makes the immune interactions between the maternal and the fetal inevitable (8–10). Bearing both maternal and paternal antigens, the fetus is regarded as semi-allografts by the maternal immune system. Therefore, an immunotolerance mechanism must exist at the maternal-fetal interface to secure a successful pregnancy, as suggested by Sir Peter Brian Medawar six decades ago (11, 12). However, the failure of this mechanism may lead to many pregnancy-related diseases, such as recurrent spontaneous abortion and PTB (7, 13–19). Unfortunately, the precise mechanism by which immune disorders contribute to these pregnancy complications is still incompletely understood and warrants further study (20).

Previous studies have been focused on characterizing immune response in the peripheral blood to uncover the underlying mechanisms of pathogenic pregnancy (21–23); however, the systemic inflammation in the periphery may be far less relevant to pregnancy than the local inflammation at the maternal-fetal interface, as suggested by a systematic review (7). The maternal-fetal interface is an important site where dynamic changes of inflammatory status and immune deviations are tightly regulated across different trimesters (24–27). The key event contributing to the unique maternal-fetal immune adaptation during pregnancy is the complex and delicate crosstalk between trophoblasts and decidual immune cells (28), which could be slightly different at different sites of the maternal-fetal interface. For example, the decidua basalis contacts with both the uterus wall and the placenta villus, and contains immune cells of both maternal and fetal origin, and it is therefore considered as the major site of the most numerous and complex maternal-fetal cellular interactions and the regulation of placenta development. Different from the decidua basalis, the decidua capsularis and parietalis fuse during late pregnancy, it comes into direct contact with the uterus wall and majorly harbors maternal-origin lymphoid and myeloid cells (29). Hence, the decidua basalis, as well as the decidua capsularis and parietalis, which are called collectively as the decidua parietalis in this article, have been the focus of many studies when exploring the immunology of the maternal-fetal interface during pregnancy (17, 18, 30).

The human immune system includes both innate and adaptive arms. Macrophages account for around 20%-30% of decidual leukocytes and are one of the major innate immune cells at the maternal-fetal interface (6, 24). These cells can be classified into two subtypes according to their distinct cytokine profiles and functionalities: M1 subtype which displays a pro-inflammatory function, and anti-inflammatory M2 subtype that is critical for tissue homeostasis (31–34). On the other hand, as a major component of the adaptive immune system, T helper (Th) cells play a vital role in mediating and regulating the immune response. These cells can also be divided into diverse functional subpopulations (e.g., Th1, Th2, Th17 and Treg) according to their cytokine profiles as well. Dysregulation of M1/M2 macrophages or Th1/Th2/Th17/Treg cells in decidua was reported in multiple pathogenic pregnancies (15, 35, 36). However, the distribution of these immune cells at the maternal-fetal interface during PTB and the mechanisms by which they drive a dominant pro-inflammatory microenvironment are still incompletely understood.

In this study, we examined the distribution of M1/M2 macrophages, Th1/Th2 cells and Th17/Treg cells at the decidua basalis and parietalis during term labor and PTB to explore the perturbations of the innate and adaptive immune response during parturition. Our study may uncover the possible mechanism of the breakdown of maternal-fetal tolerance during PTB.

## Methods

This study was conducted between October 2020 and October 2021 at Tongji Hospital, Tongji Medical College, Huazhong University of Science and Technology (Wuhan, Hubei). The institutional review board of Tongji Medical College approved the study (ID: [2020] S190; Date: Oct 13, 2020). Written informed consents were obtained from all participants.

### Participants Enrollment

This study was conducted between October 2020 and October 2021 at Tongji Hospital, Tongji Medical College, Huazhong University of Science and Technology (Wuhan, Hubei). The institutional review board of Tongji Medical College approved the study (ID: [2020] S190; Date: Oct 13, 2020). Written informed consents were obtained from all participants.

Women delivered with or without labor at term or preterm were classified into four groups: term not in labor group (TNL, N=19), term in labor group (TL, N=17), preterm not in labor group (PNL, N=10), and preterm in labor group (PIL, N=10). Labor was defined by the presence of regular uterine contractions, cervical effacement, and a descending fetus. Preterm labor was defined as delivering between 24–36^+6^ weeks of gestation, while term labor was defined as delivering between 37–41^+6^ weeks of gestation. The exclusion criteria included multiple pregnancies, obvious clinical signs of infection (e.g., body temperature > 38°C, maternal WBC count > 1.5 × 10^6^/L, foul-smelling vaginal secretion, uterine tenderness, maternal tachycardia and/or fetal tachycardia), severe hepatitis, severe preeclampsia and eclampsia. HE staining of the placenta and fetal membrane in all participants presented no signs of subclinical infections such as obvious neutrophil infiltration. Participants in the PNL groups terminated the pregnancy for placenta previa with or without placenta increta (N=4), acute fetal distress (N=2), oligohydramnios (N=2), threatened uterine rupture (N=1), placenta abruption (N=1). Clinical characteristics of the participants are presented in **Table 1**.

**Table 1:** Clinical Characteristics of Participants.

	TNL (N=19)	TL (N=17)	PNL (N=10)	PIL (N=10)	*P* value
**Maternal Age (years)** ^a^	31 (30, 34)	29 (28, 32)	32 (30, 34)	29 (28, 32)	0.092
**BMI (kg/m^2^)** ^a^	25.6 (24.7, 27.8)	26.1 (23.6, 28.1)	26.0 (24.1, 27.9)	24.9 (21.9, 29.2)	0.857
**Gravidity** ^a^	2 (1, 3)	1 (1, 2)	3 (1, 4)	2 (1, 3)	0.145
**Parity** ^a^	0 (0, 1)	0 (0, 1)	0 (0, 1)	0 (0, 1)	0.379
**Assisted Reproductive Technology**^b^	4 (21.1%)	2 (11.8%)	0 (0%)	1 (10.0%)	0.527
**Gestational Age (weeks)** ^a^	38.6 (38.2, 39.1)	39.6 (38.6, 39.8)	34.8 (32.8, 35.8)	34.4 (30.0, 36.0)	< 0.001
**Caesarean Delivery**^b^	19 (100%)	0 (0%)	10 (100%)	2 (20.0%)	< 0.001
**Birthweight (g)** ^a^	3450 (3200, 3570)	3350 (3075, 3500)	2315 (1630, 2732)	2100 (1478, 2580)	< 0.001
**Neonatal Gender**^b^					
**Male**	7 (36.8%)	8 (47.1%)	6 (60.0%)	4 (40.0%)	0.668
**Female**	12 (63.2%)	9 (52.9%)	4 (40.0%)	6 (60.0%)	
**Apgar Score** ^a^					
**1 min**	8 (8, 8)	8 (8, 8)	8 (7, 8)	8 (7, 8)	0.186
**5 min**	9 (9, 9)	9 (9, 9)	9 (9, 9)	9 (8, 9)	0.010
**Admission to Department of Neonatology**^b^	1 (5.3%)	0 (0%)	5 (50.0%)	6 (60.0%)	< 0.001

Values are given as median (interquartile range) or number (percentage), unless indicated otherwise. BMI, body mass index.

^a^Kruskal-Wallis Test; ^b^Fisher’s Exact Test.

### Tissue Preparation

**Figure 1:**
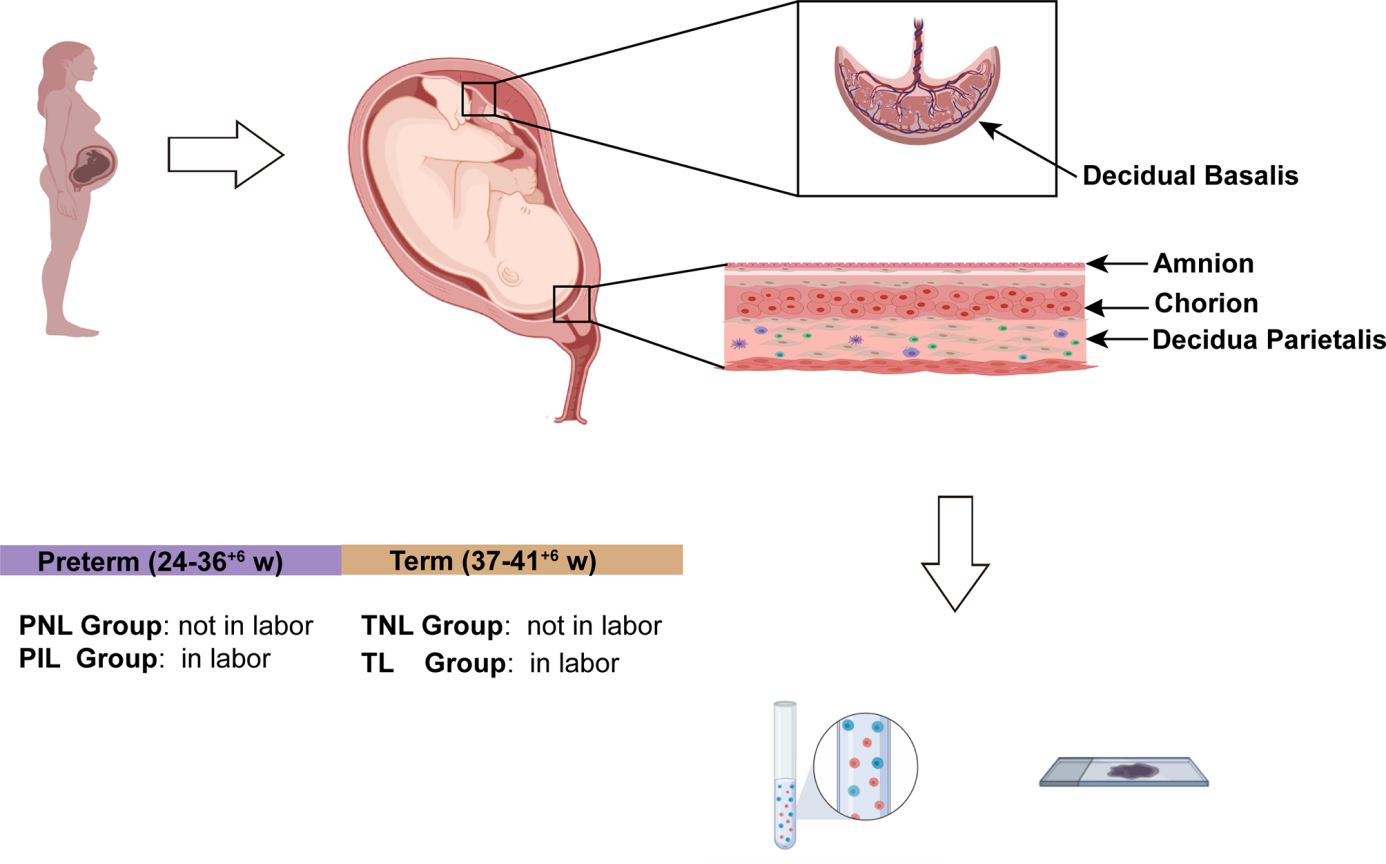
Study Design of the Experiment. The figure was created with BioRender.com (https://app.biorender.com).

Human tissues of the decidua including two compartments were collected immediately after delivery (37). In brief, the decidua basalis and parietalis were collected from basal plate and chorioamniotic membranes, respectively. Tissues were washed with ice-cold sterile phosphate buffer saline (PBS) to remove the blood and then divided into two parts (**Figure 1**). One part was fixed with polyformaldehyde, embedded in paraffin and cut into sections (3 μm) for immunohistochemical analysis, while the other part of fresh decidua basalis and parietalis (20 g) were rinsed thoroughly with sterile PBS containing 1% penicillin/streptomycin (Cat. 10378016, Gibco) to remove the blood, and were then cut and homogenized using a gentleMACS Dissociator (Miltenyi Biotec) in RPMI 1640 medium supplemented with 0.25% Trypsin (Cat. G4001, Servicebio), collagenase Type IV (Cat. G5027, Servicebio) at 1.0 mg/ml concentration, and deoxyribonuclease I (Cat. B002004, Sangon Biotech) at 0.1 mg/ml concentration (15, 37). Subsequently, homogenized tissues were digested for 60 min at 37°C with gentle agitation, then filtered through a 70 μm cell strainer (Cat. BS-70-XBS, Biosharp) and washed in PBS to obtain single-cell suspensions, which were stored in liquid nitrogen for future flow cytometry analysis.

### Flow Cytometric Analysis

Flow cytometry was performed to investigate the distribution of Th cells and macrophages in the decidua using single-cell suspensions isolated from both decidua basalis and parietalis during term labor and PTB, which were first incubated in a 5% CO_2_ incubator at 37°C resting for 1 h after resuscitation to allow the full recovery of the cells. Macrophage subsets, as well as Th1, Th2, Th17 and Treg cells were then stained using different staining panels (**Supplementary Table S1**).

For staining of macrophages, 1 × 10^6^ single-cell suspensions were washed by ice-cold staining buffer (PBS containing 2% fetal bovine serum and 0.1% azide), and stained with BV510-conjugated Fixable Viability Stain (FVS, **Supplementary Table S1****)** in the dark for 15 min at room temperature followed by incubating with Fc Receptor Blocking Solution (Cat. 422302, BioLegend) for 10 min at 4°C. Cells were then stained for surface markers with antibodies including allophycocyanin (APC)-Cy7-conjugated anti-human CD45 antibody, APC-conjugated anti-human CD11b antibody, phycoerythrin (PE)-Cy7-conjugated anti-human CD68 antibody, BV421-conjugated anti-human CD86 antibody, and PE-conjugated anti-human CD206 antibody for 30 min at 4°C in the dark. After surface staining, cells were fixed and permeabilized by the Cytofix/Cytoperm Kit (Cat. No. 554714, BD Bioscience) according to the manufacturer’s instructions, and were further stained with fluorescein isothiocyanate (FITC)-conjugated anti-human iNOS antibody at 4°C in the dark for 30 min.

For staining of Th1, Th2 and Th17 cells, 1 × 10^6^ cells were incubated with 2 μl of Leukocyte Activation Cocktail (Cat. 550583, BD Bioscience) for 6 h and then washed by ice-cold staining buffer before staining. Cells were first incubated with FITC-conjugated FVS and Fc Receptor Blocking Solution as described above, and were then stained with APC-Cy7-conjugated anti-human CD45 antibody, BV510-conjugated anti-human CD3 antibody, APC-conjugated anti-human CD4 antibody, and BB700-conjugated anti-human CD8 antibody at 4°C in the dark for 30 min. After that, cells were fixed and permeabilized as mentioned above followed by intracellular staining with PE-Cy7-conjugated anti-human IFN-γ antibody, BV421-conjugated anti-human IL-4 antibody, and PE-conjugated anti-human IL-17A antibody at 4°C in the dark for 30 min.

For staining of Treg cells, 1 × 10^6^ cells were washed once and stained by FITC-conjugated FVS and Fc Receptor Blocking Solution as mentioned above, then the cells were stained with APC-Cy7-conjugated anti-human CD45 antibody, BV510-conjugated anti-human CD3 antibody, APC-conjugated anti-human CD4 antibody, BB700-conjugated anti-human CD8 antibody, PE-conjugated anti-human CD127 antibody and BV421-conjugated anti-human CD25 antibody at 4°C in the dark for 30 min.

After staining, cells were washed once and then resuspended in 300 μl of 1% paraformaldehyde followed by flow cytometric analysis on a BD LSR Fortessa instrument (BD Bioscience). Data were all analyzed by FlowJoV10 (BD Bioscience) in this study.

### Immunohistochemistry and Immunofluorescence

IHC was used to examine the presence of M1, M2 and Th1 cells in decidua basalis and parietalis tissues *in situ*. The paraffin sections were first dewaxed and rehydrated, and were then immersed and boiled in citrate antigen retrieval solution (Cat. G1202, Servicebio, pH = 6.0) for 15 min. After the sections cooled down to room temperature, the endogenous peroxidase activity was blocked using 3% H_2_O_2_ for 15 min at 37°C, and non-specific binding was blocked by 5% bovine serum albumin (BSA) solution for 30 min at 37°C. Next, sections were incubated with mouse, rat or rabbit antibodies including those against human CD68 (Cat. GB14043, Servicebio, 1:200 diluted), iNOS (Cat. 53-5920-82, eBioscience, 1:100 diluted), CD206 (Cat. Ab252921, Abcam, 1:4000 diluted), and IFN-γ (Cat. 557844, BD Bioscience, 1:100 diluted) at 4°C overnight, respectively. On the next day, the sections were incubated with HRP-conjugated antibodies including goat anti-mouse IgG (Cat. GB23301, Servicebio, 1:200 diluted), goat anti-rat IgG (Cat. GB23302, Servicebio, 1:200 diluted) or goat anti-rabbit IgG (Cat. GB23303, Servicebio, 1:200 diluted) for 30 min at 37°C according to the primary antibodies used. After incubation with secondary antibody, the sections were incubated with 3,3-diaminobenzidine tetrahydrochloride (Cat. G1212-200T, Servicebio) for 5 min to present the HRP activity, and the nucleus was stained by hematoxylin for 5 min. Between each step, the sections were always washed three times in Tris Buffered Saline Tween (TBST, Tris-buffered saline containing 1‰ Tween-20 and 0.3‰ Triton X-100). After that, the sections were dehydrated and mounted with resinene for microscope observation.

IF was used to reveal the presence of Treg cells in decidua basalis and parietalis. As mentioned above, the paraffin sections were dewaxed and rehydrated, then the sections were boiled in citrate antigen retrieval solution (Cat. G1203, Servicebio, pH = 9.0) for 15 min. Next, non-specific binding was blocked by 5% BSA solution for 30 min. After that, the sections were incubated with mouse anti-human FOXP3 antibody (Cat. Ab20034, Abcam, 1:50 diluted) overnight at 4°C. On the next day, the sections were incubated with Cy3-conjugated goat anti-mouse IgG (Cat. GB21301, Servicebio, 1:200 diluted) for 30 min at dark, and the nucleus was stained by hematoxylin 4’,6-diamidino-2-phenylindole (DAPI, Cat. G1012, Servicebio) for 5 min at dark. The sections were washed three times between each step as described above. Finally, the sections were mounted with Fluoromount-G (Cat. 0100-01, SouthernBiotech). The DSY2000X system (UOP, Chongqing, China) was used to gain the images of the IHC and IF sections, each section was observed in five different fields. ImageJ (Version 1.53a) was used for the quantitative measurement of each IHC and IF results, the integrated density (IntDen)/Area was used to calculate the results and the average of five different fields of each section were taken for comparison.

### Statistical Analysis

SPSS (Version 26.0, IBM) was used for general statistical analysis and GraphPad Prism (Version 8.4.0) was used to plot the data. Continuous variables were presented as median (interquartile range) or mean ± standard deviation (SD). Depending on the distribution of continuous variables, Two-tailed Students’ *t*-test or Mann-Whitney *U* test was used for comparison between two groups, and one-way analysis of variance or Kruskal-Wallis test was used for comparison among three or more groups. Paired *t-*test or Wilcoxon matched-pairs signed ranks test was used for comparison for paired variables between two groups. Categorical variables were presented as numbers (percentage) and compared using *χ*^2^ test or Fisher’s exact test. Differences were considered statistically significant when the *P* value was less than 0.05.

## Results

### Decreased Frequencies of M2 Macrophage and Increased M1/M2 Ratio in The Decidua of Preterm Labor Patients but Not Term Labor Subjects

**Figure 2:**
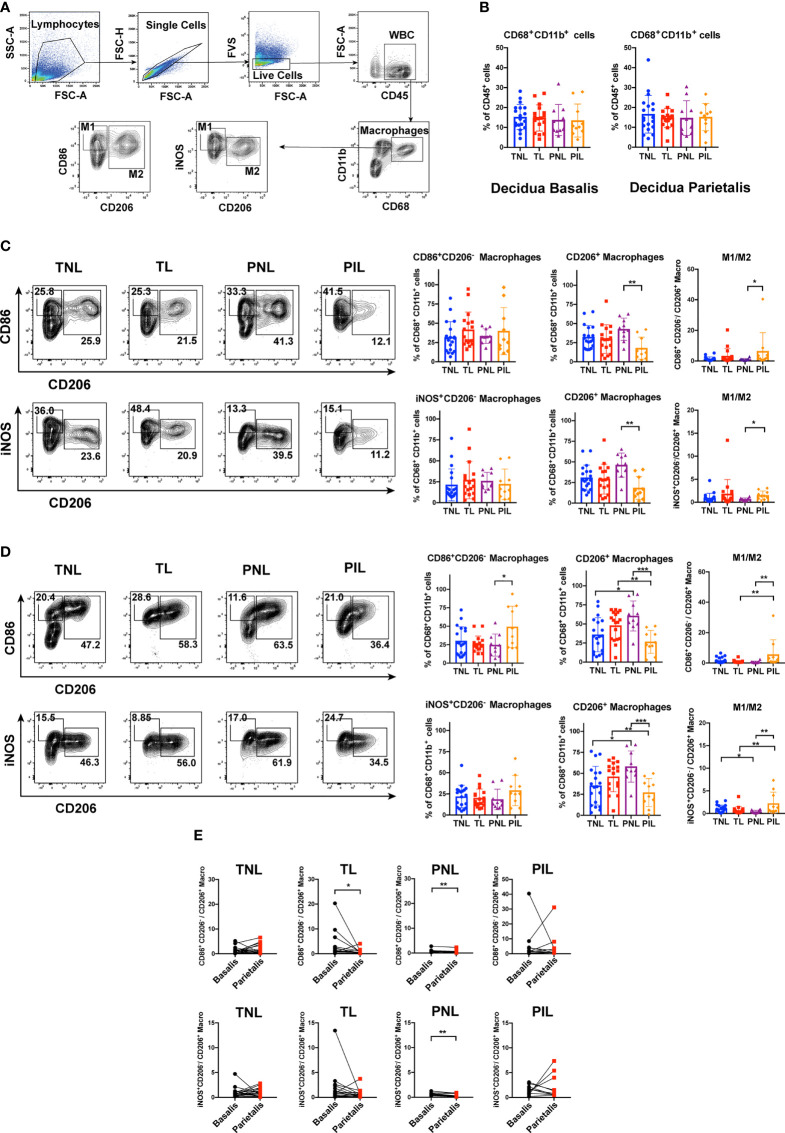
Flow Cytometric Analysis of Macrophages in the Decidua basalis and Parietalis during Term and Preterm Labor. **(A)** Gating strategy used for determining macrophages (CD45^+^CD68^+^CD11b^+^ cells), M1 (CD86^+^CD206^-^ cells or iNOS^+^CD206^-^ cells) and M2 (CD206^+^ cells); **(B)** Comparison of the frequencies of total macrophages in CD45^+^ cells among the four groups in the decidua basalis and parietalis; **(C)** Comparison of the frequencies of M1 and M2 macrophages, as well as the M1/M2 ratio among the four groups in the decidua basalis; **(D)** Comparison of the frequencies of M1 and M2 macrophages, as well as the M1/M2 ratio among the four groups in the decidua parietalis; **(E)** Comparison of the M1/M2 ratio between the decidua basalis and parietalis among the four groups respectively. Continuous variables were presented as mean ± standard deviation. Mann-Whitney U test was used for comparison between two groups, Wilcoxon matched-pairs signed rank test were used for comparison between decidua basalis and parietalis. Differences are indicated: * *P* < 0.05, ** *P* < 0.01 and *** *P* < 0.001. TNL group, N = 19; TL group, N = 17; PNL group, N = 10; PIL group, N = 10.

Macrophages are broadly categorized into M1 and M2 subsets and there is mounting evidence indicating that macrophage polarization plays a pivotal role in immune response at the maternal-fetal interface during pregnancy (15, 31). However, the role of macrophages in preterm labor has not been specifically addressed. Thus, we sought to investigate the distribution of macrophages and their subsets in decidua basalis and parietalis during term and preterm labor using flow cytometry. The gating strategy for decidual macrophages was shown in **Figure 2A**. Macrophages were determined as CD45^+^CD68^+^CD11b^+^ cells, while M1 and M2 macrophage subtypes were defined as CD45^+^CD68^+^CD11b^+^CD86^+^CD206^-^ or CD45^+^CD68^+^CD11b^+^iNOS^+^CD206^-^, and CD45^+^CD68^+^CD11b^+^CD206^+^ cells, respectively. The FMO controls of M1- and M2-related markers were presented in **Supplementary Figure 1**. We first compared the relative frequencies of total macrophages in all leukocytes (CD45^+^ cells). Our results indicated that the frequencies were all comparable in the four groups (i.e., TNL, TL, PNL and PIL) in both decidua basalis and parietalis (**Figure 2B**).

We next examined the frequencies of macrophage subsets in total macrophages. In decidua basalis, the frequencies of M1 cells were comparable in the four groups when they were determined as either CD86^+^CD206^-^ (**Figure 2C****, upper panel**) or iNOS^+^CD206^-^ (**Figure 2C****, lower panel**). However, the frequencies of CD206^+^ M2 cells in total macrophages were significantly decreased in the PIL group compared to that in the PNL group (*P* < 0.01, **Figure 2C**), and not surprisingly, resulted in a significantly increased M1/M2 ratio in the PIL group compared to that in the PNL group (*P* < 0.05, **Figure 2C**).

In decidua parietalis, there was no statistical difference between the TNL and TL groups in terms of the frequencies of M1 cells determined by either CD86^+^CD206^-^ or iNOS^+^CD206^-^, and M2 cells (**Figure 2D**), which is similar to what we observed in decidua basalis. In the PIL versus PNL comparison, the frequencies of M2 cells were also significantly decreased (*P* < 0.001) as noted in decidua basalis tissue, whereas increased frequencies of M1 cells were observed only when these cells are defined as CD86^+^CD206^-^ (*P* = 0.030) but not iNOS^+^CD206^-^. Nonetheless, the M1/M2 ratio was similarly increased in the PIL group compared to that in the PNL group in this tissue (*P* < 0.01). Interestingly, we found that the TNL group had lower frequencies of CD206^+^ M2 cells and higher M1/M2 ratios than the PNL group, while the TL group had higher frequencies of CD206^+^ M2 cells and lower M1/M2 ratios than the PIL group (*P* < 0.05) (**Figure 2D**). Of note, the M1/M2 ratio appeared to be higher in decidua basalis compared to that in decidua parietalis, especially in the PNL group, indicating the differential polarization status in different tissues (**Figure 2E**, and will discuss later).

**Figure 3:**
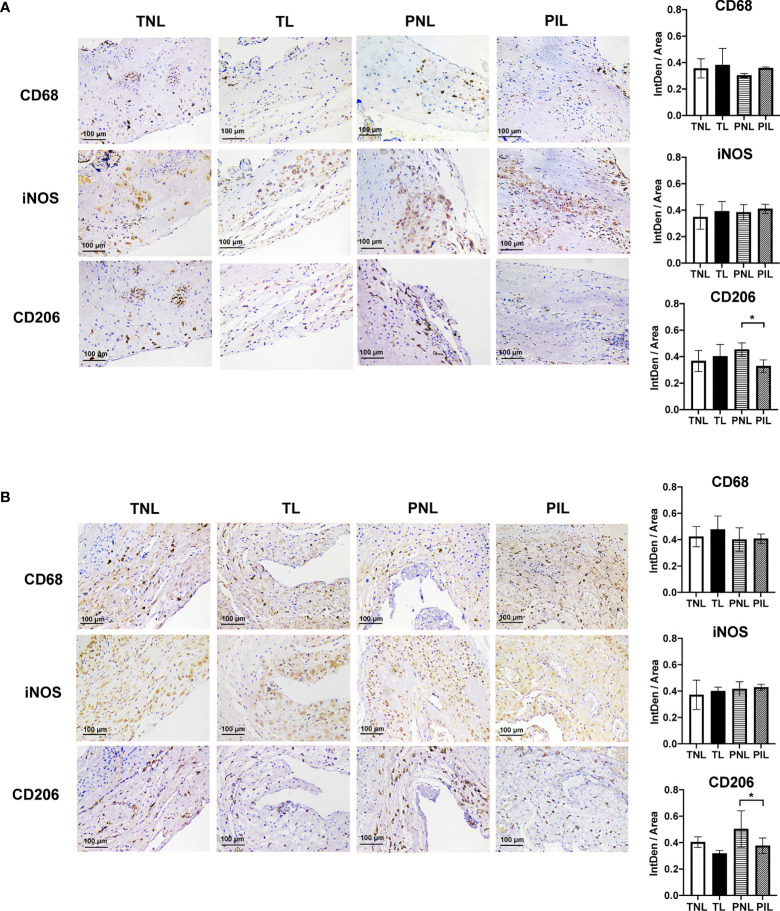
IHC Staining of Macrophages Markers in the Decidua basalis and Parietalis during Term and Preterm Labor. **(A)** Comparison of the expression of CD68, iNOS, and CD206 in the decidua basalis tissue among the four groups. **(B)** Comparison of the expression of CD68, iNOS, and CD206 in the decidua parietalis tissue among the four groups. Continuous variables were presented as mean ± standard deviation. Differences are indicated: * *P* < 0.05. IntDen: Integrated density.

To verify the above findings *in situ*, we used IHC to reveal the decidual M1 and M2 macrophages by staining CD68, iNOS and CD206 in the paraffin sections of the decidua basalis and parietalis. Similar to our results in flow cytometry, decreased expression of CD206 in the PIL group compared with the PNL group in both decidua basalis and parietalis were noted (*P* < 0.05), but the expression of CD68 and iNOS did not present a statistical difference between the two groups (**Figure 3**). Moreover, no significant difference was found between the TNL and TL groups regarding the expression of these macrophage markers in both decidua basalis and parietalis. In summary, our results indicated that preterm labor patients but not term labor subjects have significantly decreased frequencies of M2 cells and increased M1/M2 ratio in the decidua compared to not in labor controls.

### Elevated Th1 Cells and Increased Th1/Th2 Ratio in The Decidua of Term Labor Subjects but Not Preterm Labor Patients

**Figure 4:**
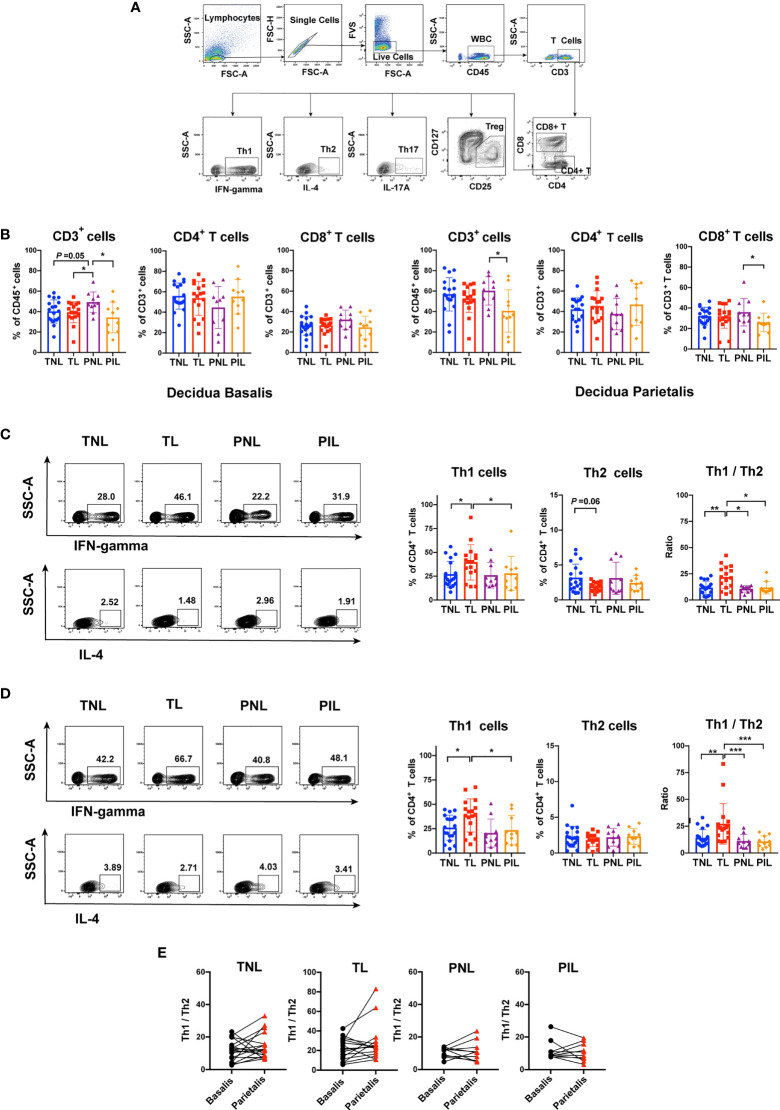
Flow Cytometric Analysis of Th1 and Th2 cells in the Decidua basalis and Parietalis during Term and Preterm Labor. **(A)** Gating strategy used for determining T cells (CD45^+^CD3^+^ cells), CD4^+^ T cells, CD8^+^ T cells, Th1 cells (IFN-γ^+^CD4^+^cells), Th2 (IL-4^+^CD4^+^ T cells), Th17 (IL-17A^+^CD4^+^ T cells) and Treg (CD25^high^CD127^low^CD4^+^ T cells); **(B)** Comparison of the frequencies of CD3^+^cell in CD45^+^ cells, and the percentages of CD4^+^ T cells and CD8^+^ T cells in total T cells among the four groups in the decidua basalis (left) and parietalis (right) tissues; **(C)** Comparison of the frequencies of Th1 and Th2 cells as well as Th1/Th2 ratio among the four groups in the decidua basalis; **(D)** Comparison of the frequencies of Th1 and Th2 cells as well as Th1/Th2 ratio among the four groups in the decidua parietalis; **(E)** Comparison of Th1/Th2 ratio between the decidua basalis and parietalis among the four groups respectively. Continuous variables were presented as mean ± standard deviation. Mann-Whitney U test was used for comparison between two groups, Wilcoxon matched-pairs signed rank test were used for comparison between decidua basalis and parietalis. Differences are indicated: * *P* < 0.05, ** *P* < 0.01 and *** *P* < 0.001. TNL group, N = 19; TL group, N = 17; PNL group, N = 10; PIL group, N = 10.

To assess the distribution of T cells in the decidua, we examined the frequencies of T cells and their functional subsets using flow cytometry with the gating strategy shown in **Figure 4A**. We first compared the frequencies of all T cells (CD3^+^ cells) in leukocytes (CD45^+^ cells), the percentages of CD4^+^ T cells (T helper cells) and CD8^+^ T cells (cytotoxic T cells) in total T cells among the four groups in both decidua basalis and parietalis. In the decidua basalis, the PNL group presented a higher frequency of total T cells than the PIL group (*P* = 0.025), the TNL group (*P* = 0.050), and the TL group (*P* = 0.045), whereas the frequencies of CD4^+^ T cells and CD8^+^ T cells in decidua basalis were comparable among the four groups (**Figure 4B****, left panel**). In the decidua parietalis, the PNL group similarly presented a tendency of a higher percentage of CD3^+^ T cells (*P* = 0.029) as well as CD8^+^ T cells (*P* = 0.030) than the PIL group (**Figure 4B****, right panel**).

We further compared the frequencies of Th1 cells (IFN-γ^+^ CD4^+^ T cells) and Th2 cells (IL-4^+^ CD4^+^ T cells) during term and preterm labor. Compared with the TNL group, the TL group had significantly elevated percentages of Th1 cells in both decidua basalis (*P* = 0.047) and parietalis (*P* = 0.020) (**Figures 4C, D****)**. In decidua basalis, the frequency of Th2 cells in the TL group tended to be lower than that in the TNL group, but the difference was not significant (*P* = 0.060) (**Figure 4C**). We next calculated and compared the ratio of Th1 and Th2 cells among the four groups, the results showed that the TL group had the highest ratio of Th1/Th2 in both decidua basalis and parietalis, which was significantly higher than the TNL group (*P* < 0.01), the PNL group (*P* < 0.05), and the PIL group (*P* < 0.05) (**Figures 4C, D****)**. However, such differences were not observed between the preterm groups, in which the PIL group presented neither elevated frequency of Th1 cells nor increased Th1/Th2 ratio compared to the PNL group (**Figures 4C, D****)**. When the Th1/Th2 ratio between the decidua basalis and parietalis in the four groups was compared, no statistical difference was found (**Figure 4E**).

**Figure 5:**
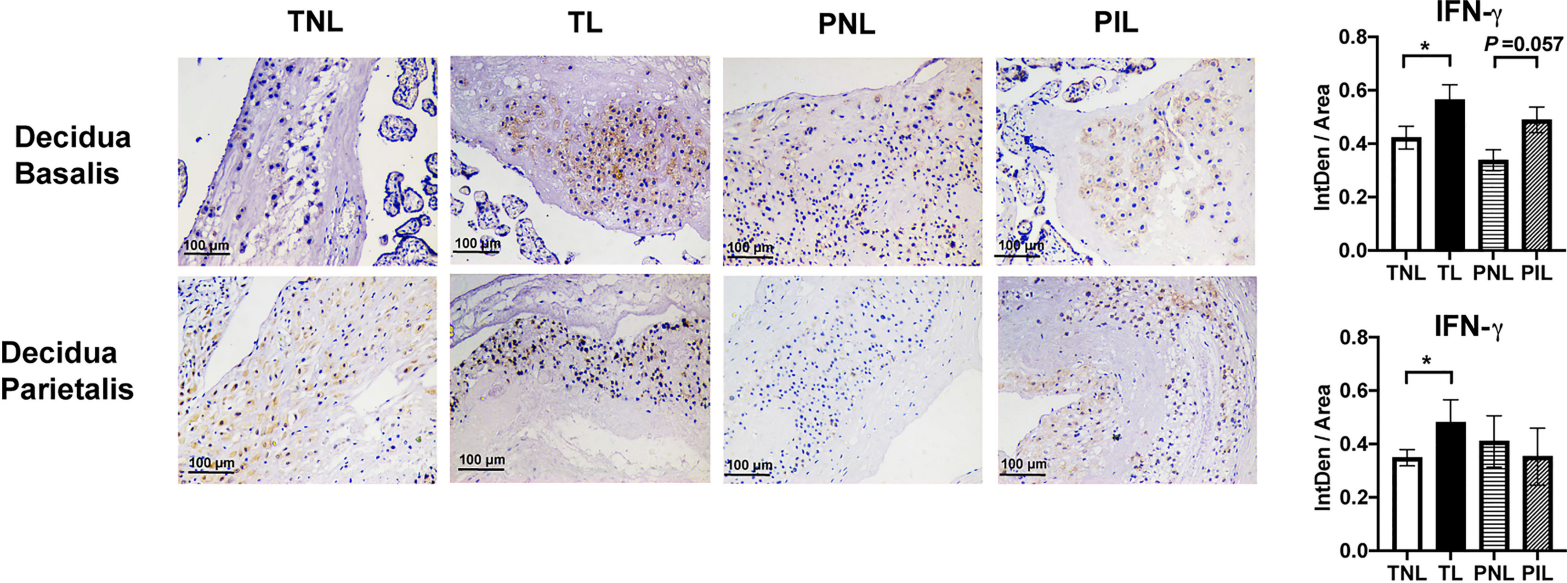
IHC Staining of IFN-γ in the Decidua basalis and Parietalis during Term and Preterm Labor. Comparison of the expression of IFN-γ in the decidua basalis and parietalis among the four groups. Continuous variables were presented as mean ± standard deviation. Differences are indicated: * *P* < 0.05. IntDen: Integrated density.

To verify the elevated frequency of the Th1 cells during term labor *in situ*, we used IHC to reveal the Th1 bias by staining IFN-γ (a Th1 cytokine) in the paraffin samples of the decidua basalis and parietalis. As shown in **Figure 5**, both the decidua basalis and parietalis samples of the TL group presented higher expression of IFN-γ compared with that of the TNL group (*P* < 0.05). In the PIL versus PNL comparison, only a slightly higher level of IFN-γ was observed in the PIL group than the PNL group (*P* = 0.057) in decidua basalis but not in decidua parietalis. Together, these results demonstrated that increased Th1 cells and Th1/Th2 ratio are more prominent in the decidua of term labor subjects but not preterm labor patients.

### Elevated Th17/Treg Ratio in The Decidua Basalis Than Parietalis During the Term and Preterm Labor

**Figure 6:**
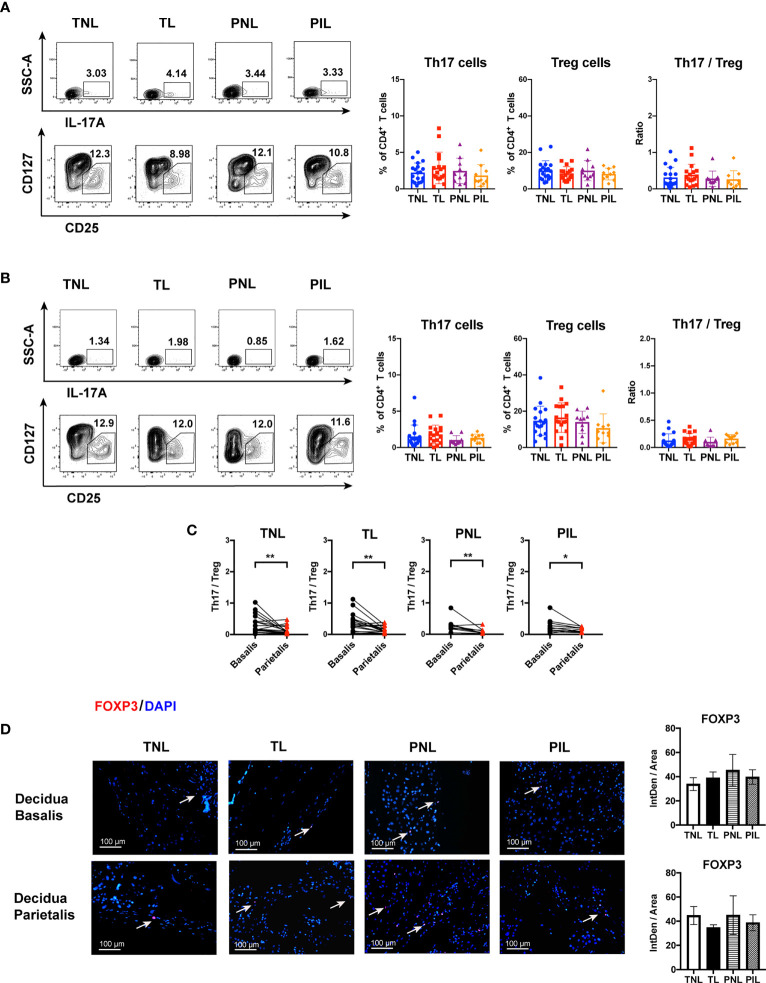
Flow Cytometric Analysis of Th17 and Treg cells, and IF Staining of FOXP3 in the Decidua basalis and Parietalis during Term and Preterm Labor. **(A)** Comparison of the frequencies of Th17 and Treg cells as well as Th17/Treg ratio among the four groups in the decidua basalis; **(B)** Comparison of the frequencies of Th17 and Treg as well as Th17/Treg ratio among the four groups in the decidua parietalis; **(C)** Comparison of Th17/Treg ratio between the decidua basalis and parietalis among the four groups respectively; **(D)** Comparison of the expression of FOXP3 (Red) in the decidua basalis and parietalis among the four groups, the nuclei were stained with DAPI (blue) and the positive staining was indicated by the white arrow. Continuous variables were presented as mean ± standard deviation. Mann-Whitney U test was used for comparison between two groups, Wilcoxon matched-pairs signed rank test were used for comparison between decidua basalis and parietalis. Differences are indicated: *P < 0.05, **P < 0.01. TNL group, N = 19; TL group, N = 17; PNL group, N = 10; PIL group, N = 10. IntDen: Integrated density.

Naive CD4^+^ T cells can differentiate into Th17/Treg precursor cells which may further differentiate into either Th17 or Treg cells under certain cytokine milieu. The balance of Th17 and Treg cells was reported to be critical in determining the outcome of pregnancy (36, 38). Therefore, we also investigated the frequencies of Th17 and Treg cells, as well as the Th17/Treg ratio in the decidua. As shown in **Figures 6A, B**, the frequencies of Th17 cells (IL-17A^+^CD4^+^ T cells) and Treg cells (CD25^high^CD127^low^CD4^+^ T cells) in decidua basalis and parietalis were comparable among the four groups, so was the ratio of Th17 cells and Treg cells. Interestingly, the decidua basalis had a significantly higher Th17/Treg ratio than the decidua parietalis in the TNL, TL, PNL and PIL groups (*P* < 0.05) (**Figure 6C**, and will discuss later).

Considering the low frequency of Th17 cells in the decidua as indicated by flow cytometry, we alternatively verify the above results through staining for the expression of FOXP3 to reveal the presence of Treg in decidua basalis and parietalis by IF. As expected, the expression of FOXP3 can be detected by IF in both decidua basalis and parietalis, but no difference was found among the four groups in different tissues (**Figure 6D**).

## Discussion

Pregnancy is a marvelous process in which the maternal immune system must be tolerant to the hemi-allogeneic fetus while maintaining the capability of resisting harmful pathogens. The maternal-fetal interface is an area where numerous maternal, fetal and placental cells in close contact with each other, such intimate contact leads to tightly controlled immune interactions between the mother and the fetus, and thus protects the fetus from immunological rejection by the maternal immune system (8). In fact, these complex interactions are necessary for modulating the microenvironment to secure a successful pregnancy. Recent studies have found that precisely tuned immune adaptation, the so-called “immune clock”, plays a vital role in pregnancy maintenance and triggering the onset of birth (20, 39), whereas dysregulations of the immune clock were increasingly recognized as a potential cause of many pregnancy complications including PTB (18, 39, 40). Therefore, elucidating immunological cross-talk at the maternal-fetal interface during term labor and PTB has always been a subject of substantial interest in obstetric research. In order to get a better understanding of the unique immunological features at the maternal-fetal interface and their dynamic changes during parturition, we compared the frequencies of the major immune cell subsets including macrophages, Th cells, and Treg cells in the decidua (i.e., decidua basalis and parietalis) between term and PTB in this study. The major findings of this study can be summarized as follows: i) the PIL group has significantly elevated M1/M2 ratio and comparable Th1/Th2 ratio than the PNL group; ii) the TL group has an increased Th1/Th2 ratio but not M1/M2 ratio compared to the TNL group; iii) the Th17/Treg ratio in decidua basalis was higher than that in decidua parietalis among the four groups.

Macrophages are among the primary innate immune cells in the decidua that are implicated in the process of pregnancy (6, 10, 41). In mouse models of PTB induced by inoculating intravaginally with lipopolysaccharide or progesterone antagonist RU486, Gonzalez et al. observed increased macrophage-mediated remodeling of the cervix (42). More strikingly, Pique-Regi et al. recently found that the most differentially expressed genes in the decidua during the term and preterm labor were observed in the macrophages. These studies highlight the importance of macrophages during PTB (29). However, the distribution of macrophages and their subsets in PTB is less characterized. Gomez-Lopez and colleagues reported that women at preterm with spontaneous labor (PIL) had elevated M1 macrophages, comparable frequencies of M2 macrophages and accordingly higher M1/M2 ratio in decidua basalis compared with those at preterm without spontaneous labor (PNL), and had similar frequencies of both macrophage subtypes when compared to women at term with spontaneous labor (TL) (17). However, the latter result was overturned by the same group in another study when using more samples, in which the PIL group was found to have reduced M2 macrophages and increased TNF expression in proinflammatory M1 macrophages than the TL group in both decidua basalis and parietalis (18). In this study, we also found that the PIL group had increased M1/M2 ratio in the decidua when compared with the PNL group or the TL group (**Figures 2**, **3**), corroborating previously published data (17, 18). Of note, the frequencies of M1 and M2 cells are different in these studies, these discrepancies may result from the distinct criteria selected for defining these cells: CD45^+^CD14^+^ICAM3^−^CD80^+^ and CD45^+^CD14^+^ICAM3^−^CD209^+^CD163^+^ cells were used by Gomez-Lopez and colleagues, whereas CD45^+^CD68^+^CD11b^+^CD86^+^CD206^−^ or CD45^+^CD68^+^CD11b^+^iNOS^+^CD206^−^ and CD45^+^CD68^+^CD11b^+^CD206^+^ were used by us to define M1 and M2 cells, respectively (15, 17, 18). Nevertheless, these findings collectively suggest that M1/M2 macrophages paradigm shift plays a pivotal role in the dysregulated immune deviation in the decidua during PTB. Although the M1 and M2 percentages as well as M1/M2 ratio in our study presented no significant difference between the TNL and TL groups, we observed that the TL group has a tendency of increased M1/M2 ratio than the TNL group in decidua basalis (0.60 *vs*. 0.90, *P* = 0.27, when M1 cells were determined as iNOS^+^CD206^−^; 1.10 *vs*. 1.22, *P* = 0.29, when M1 cells were determined as CD86^+^CD206^−^) (**Figure 2**). These results were partially in agreement with the single-cell RNA-seq data by Pique-Regi et al., in which the expression of *NFKB1* by maternal macrophages at the maternal-fetal interface was found to be higher in the TL group than the TNL group. Together, the M1 bias and the resulting elevated pro-inflammatory cytokine production might be important features of term labor, and are more prominent during preterm labor (29).

Previous studies indicated that maternal circulating T cells infiltrate into the maternal-fetal interface and contribute to the inflammatory microenvironments at the maternal-fetal interface during labor (43, 44). In this study, we compare the frequencies of Th1 and Th2 cells, as well as the Th1/Th2 ratio during term labor and PTB. Our flow cytometric results showed that the TL group had obviously increased Th1 cell frequency and elevated Th1/Th2 ratio in both decidua basalis and parietalis, which were further supported by the higher expression of IFN-γ (a Th1 cytokine) in the TL group compared to that in the TNL group, as revealed by IHC staining of the decidua basalis and parietalis (**Figures 4**, **5**). However, this Th1/Th2 paradigm shift was not significant between the PIL and PNL groups. We also noticed that the TL group has the highest Th1/Th2 ratio among the four groups. Taken together, these results indicate that Th1/Th2 imbalance may be more prominent in term labor than that in PTB.

Recently, the Th1/Th2 paradigm has been expanded to include the balance of Th1, Th2, Th17 and Treg cells (36). It was reported that Th17 cells congregate in the human decidua during pregnancy, and their frequencies in Th cells were reported to be higher in the decidua compared to that in the peripheral blood (45). Th17 cells play a critical role in the induction of inflammation and fighting against extracellular microbes during normal pregnancy, but excessive Th17 immunity may also induce uncontrolled neutrophil infiltration at the maternal-fetal interface and result in pathogenic pregnancy (36, 38), highlighting the complex role of Th17 cells in pregnancy. Interestingly, the IL-7/IL-7R pathway which was reported functional on up-regulating Th17 immunity is also able to downregulate Treg immunity in recurrent spontaneous abortion (46). Treg cells are known as suppressor T cells which are involved in limiting immune response at the setting of transplantation, autoimmune diseases, and also pathogenic pregnancy. The frequency of Tregs varies throughout gestation and is altered in aberrant pregnancy (41, 47–51). Tilburgs et al. reported significantly higher percentages of CD4^+^CD25^bright^ cells in the decidua compared to that in the peripheral blood, and the proportion of those cells in the decidua significantly increased during the third trimester (52). Moreover, some studies found that Treg cells may be more important in maternal-fetal immune tolerance during the early pregnancy stage than late pregnancy stage (53–55); however, the role of Treg cells in late gestation was less pursued. To fill this knowledge gap and in an effort to unravel the role of Th17 cells and Tregs during late pregnancy, we explored in this study the distribution of these cells in the decidua during term and preterm labor. Our results showed that the frequencies of Th17 and Treg cells, as well as the ratio of them, were comparable among the four groups in both decidua basalis and parietalis (**Figure 6**). These data are partially in agreement with a previous study by Gomez-Lopez et al. in which no significant difference was found between the PIL group and the PNL group, as well as between the TL group and TNL group, in terms of the frequencies of Treg and Th17 cells in both decidua basalis and parietalis. It is worthy to note that preterm in labor women with acute or chronic inflammatory lesions of the placenta in the same study presented significantly reduced Treg cells than preterm non-labor controls (30). Therefore, although dysregulated Th17 and Treg cells appear to be less involved in preterm labor in most circumstances as demonstrated by us and Gomez-Lopez et al., they could contribute to the pathogenesis of preterm labor complicated with inflammation (30).

The M1/M2 and/or Th1/Th2/Th17/Treg paradigm shift may result in a pro-inflammatory immune environment at the maternal-fetal interface characterized by the activation of certain inflammatory pathways (e.g., MAPK pathway and NF-κB pathway) and the production of pro-inflammatory cytokines (e.g., IFN-γ, IL-1β, IL-6 and IL-8), thereby leading to the onset of both term and preterm labor. In support of this notion, markedly elevated inflammatory mediators (e.g., IL-1β, IL-6 and IL-8) were found at the maternal-fetal interface at labor onset (56). Although adaptive immune disorders such as Th1 polarization are an important feature of term labor, as indicated by our findings (**Figures 4**, **5**) and others (25, 27, 57), whether innate or adaptive immune disorders are more prominent in preterm labor is still an open question. We found in this study that patients with spontaneous preterm labor have significantly higher M1/M2 ratio but not Th1/Th2 or Th17/Treg ratio than the PNL and TL groups, suggestive of the critical contribution of polarized macrophages and dysregulated innate response to preterm labor. This notion is in agreement with a previous meta-analysis of maternal and fetal transcriptomic data, which showed the upregulation of innate immunity and the downregulation of adaptive immunity in PTB women (16). Many factors may contribute to differential profiles of immune cells between term labor and PTB. There were dynamic changes in the proportions of immune cells at the maternal-fetal interface during different trimesters, thus the lower gestational ages of women with PTB could result in the different profiles of the immune cells compared to those with term labor (24). Additionally, differential progesterone and/or estrogen levels in different gestational ages might also contribute. Progestogens and estrogen were found to influence the production of some Th1/Th17 inflammatory cytokines, and progesterone was also reported to have profound dampening effects on T cell activation and the ability to selectively regulate the expression of different genes associated with alternative macrophage activation (58–60). However, further studies with more samples and multiparameter analysis are required to examine these issues.

In this study, the decidua but not the peripheral blood was selected for characterizing immune deviations during term labor and PTB giving the higher relevance of this tissue to pregnancy. As a specialized, highly modified tissue formed by uterine endometrium during pregnancy, the decidua may act as an interaction hub of maternal-fetal cell signaling. In support of this notion, a large number of interactions between decidual cells and the syncytiotrophoblast in the cell communication network of the maternal-fetal interface were illustrated by recent single-cell transcriptome analyses of the maternal-fetal interface during early and late pregnancy (61, 62). However, the decidua is not a homogeneous tissue and can be classified into three parts including decidua basalis, decidual capsularis, and decidua parietalis. During the last trimester, the expanding sac is enlarged and completely fills the whole uterine cavity, which results in the apposition of the decidua capsularis and parietalis (63). Unfortunately, only a few studies examined the distribution of innate and adaptive immune cells in different parts of the decidua. We found in this study that the M1/M2 ratio is much higher in decidua basalis than parietalis in the TL and PNL groups, whereas the Th17/Treg ratio is unanimously higher in decidua basalis than parietalis in all groups compared (**Figures 2****–** **6**). The differential polarization status of macrophages and Th cells in different parts of the decidua (i.e., decidua basalis and parietalis) is interesting. In fact, different parts of the decidua contain distinct compositions of immune cells of either maternal or fetal origin. Using single-cell RNA-seq technique, Pique-Regi et al. demonstrated that the placental villi contained more fetal than maternal immune cells, the decidua parietalis largely contained maternal-origin lymphoid and myeloid cells, while the decidua basalis contained immune cells of both maternal and fetal origin (29). Therefore, the decidua basalis is the key site of the numerous and complex maternal-fetal cellular interactions at the maternal-fetal interface. And the differential immune deviation in decidua basalis and parietalis found by us may result from the different compositions of fetal and maternal immune cells, and the more obvious pro-inflammation response at the decidua basalis might be the result of the co-regulation of both the maternal and the fetal. However, further investigations are warranted to test this hypothesis.

The present study has several limitations, one of the limitations is the small sample size which is mainly due to the difficulty in collecting the decidua samples of PTB women. Secondly, the frequencies and functions of the non-M1 and non-M2 macrophages, as well as further subpopulations of macrophages (e.g., M2a, M2b, M2c) in the decidua of women with term or preterm labor could also be investigated. Finally, our study did not evaluate the circulating immune populations of those participants. Thus, further exploration with a larger sample size is needed in the future studies.

In conclusion, through examining the distribution of macrophage and Th cell subsets in the decidua during term labor and PTB, we found significantly elevated M1/M2 ratio during preterm labor, and increased Th1/Th2 ratio during term labor. These results suggest that the M1/M2 imbalance is probably associated with the breakdown of maternal-fetal tolerance during PTB, while the aberrant Th1/Th2 profile plays a more important role in term labor. Furthermore, we also found that the Th17/Treg ratio in decidua basalis was higher than that in decidua parietalis, indicating the more remarkable Th17/Treg imbalance in decidua basalis than parietalis. Thus, the dysregulated M1/M2 ratio during PTB, the Th1/Th2 imbalance during term labor, and the different grades of immune deviations in decidua basalis and parietalis, collectively indicate that innate and adaptive immune deviations might be differentially required for the occurrence of term labor and PTB at different locations of the maternal-fetal interface. These findings may lead to a better understanding of the pathogenesis of PTB, and will help to explore possible new strategies for the prevention and treatment of PTB.

## Data Availability Statement

The original data presented in the study are included in the article/**Supplementary Material**. Further inquiries can be directed to the corresponding authors.

## Ethics Statement

The studies involving human participants were reviewed and approved by the institutional review board of Tongji Medical College, Huazhong University of Science and Technology (ID: [2020] S190; Date: Oct 13, 2020).

## Author Contributions

WZ, YH, YZ and HL designed the study and wrote the manuscript. YZ and HL contributed equally to this work. In detail, YZ and HL are responsible for collecting the samples and performing the experiments. YH, YZ, LY and MW took the responsibilities for analyzing the data. XL, PG, YL, XG, XB, PZ, QK, XD and JW participated in collecting the samples and clinical information. WZ, LF and FQ provided access to clinical information. All authors made essential contributions to the analysis and interpretations of the results. And all authors reviewed the manuscript critically and approved the submitted version.

## Funding

This study was funded by the Natural Science Foundation of Hubei Province (2019CFB546 to XL) and the Natural Science Foundation of Xinjiang Uygur Autonomous Region (2021D01F20 to XG).

## Conflict of Interest

The authors declare that the research was conducted in the absence of any commercial or financial relationships that could be construed as a potential conflict of interest.

## Publisher’s Note

All claims expressed in this article are solely those of the authors and do not necessarily represent those of their affiliated organizations, or those of the publisher, the editors and the reviewers. Any product that may be evaluated in this article, or claim that may be made by its manufacturer, is not guaranteed or endorsed by the publisher.

## Supplementary Material

Supplementary Table 1 Staining Panels for Flow Cytometric Analyses.

Supplementary Figure 1 FMO Controls of CD86, iNOS and CD206 in Flow Cytometric Analysis of Macrophages. **(A)** FMO Controls of CD86; **(B)** FMO Controls of iNOS; **(C)** FMO Controls of CD206.
