# Supplementary material for: Immune Deviation in the Decidua During Term and Preterm Labor
Source: Front Immunol. 2022 Jun 10;13:877314. doi: 10.3389/fimmu.2022.877314 (PMC9226582; doi:10.3389/fimmu.2022.877314)
Supplement: Supplementary Table 1 — Staining Panels for Flow Cytometric Analyses. [file Table_1.docx]

| **Supplementary Table 1. Staining Panels for Flow Cytometric Analyses** | | | | |
| --- | --- | --- | --- | --- |
| **Antibodies** | **Fluorochrome** | **Source** | **Clone** | **Material Number** |
| **Panel 1** |  |  |  |  |
| Fixable Viability Stain | BV510 | BD Pharmingen | - | 564406 |
| Anti-Human CD45 | APC-CY7 | BD Pharmingen | 2D1 | 557833 |
| Anti-Human CD68 | PE-CY7 | BD Pharmingen | Y1/82A | 565595 |
| Anti-Human CD11b | APC | BD Pharmingen | ICRF44 | 550019 |
| Anti-Human CD86 | BV421 | BD Pharmingen | 2331(FUN-1) | 562432 |
| Anti-Human CD206 | PE | BD Pharmingen | 19.2 | 555954 |
| Anti-Human iNOS | FITC | eBioscience | CXNFT | 53-5920-82 |
| **Panel 2** |  |  |  |  |
| Fixable Viability Stain | FITC | BD Pharmingen |  | 564407 |
| Anti-Human CD45 | APC-Cy7 | BD Pharmingen | 2D1 | 557833 |
| Anti-Human CD3 | BV510 | BD Pharmingen | HIT3a | 564713 |
| Anti-Human CD4 | APC | BD Pharmingen | RPA-T4 | 555349 |
| Anti-Human CD8 | BB700 | BD Pharmingen | RPA-T8 | 566452 |
| Anti-Human IFN-γ | PE-Cy7 | BD Pharmingen | 4S.B3 | 557844 |
| Anti-Human IL-4 | BV421 | BD Pharmingen | MP4-25D2 | 564110 |
| Anti-Human IL-17A | PE | BD Pharmingen | SCPL1362 | 560436 |
| **Panel 3** |  |  |  |  |
| Fixable Viability Stain | FITC | BD Pharmingen | - | 564407 |
| Anti-Human CD45 | APC-Cy7 | BD Pharmingen | 2D1 | 557833 |
| Anti-Human CD3 | BV510 | BD Pharmingen | HIT3a | 564713 |
| Anti-Human CD4 | APC | BD Pharmingen | RPA-T4 | 555349 |
| Anti-Human CD8 | BB700 | BD Pharmingen | RPA-T8 | 566452 |
| Anti-Human CD25 | BV421 | BD Pharmingen | M-A251 | 562442 |
| Anti-Human CD127 | PE | BD Pharmingen | hIL-7R-M21 | 557938 |
