# Supplementary figures and images for: Immune Deviation in the Decidua During Term and Preterm Labor
Source: Front Immunol. 2022 Jun 10;13:877314. doi: 10.3389/fimmu.2022.877314 (PMC9226582; doi:10.3389/fimmu.2022.877314)

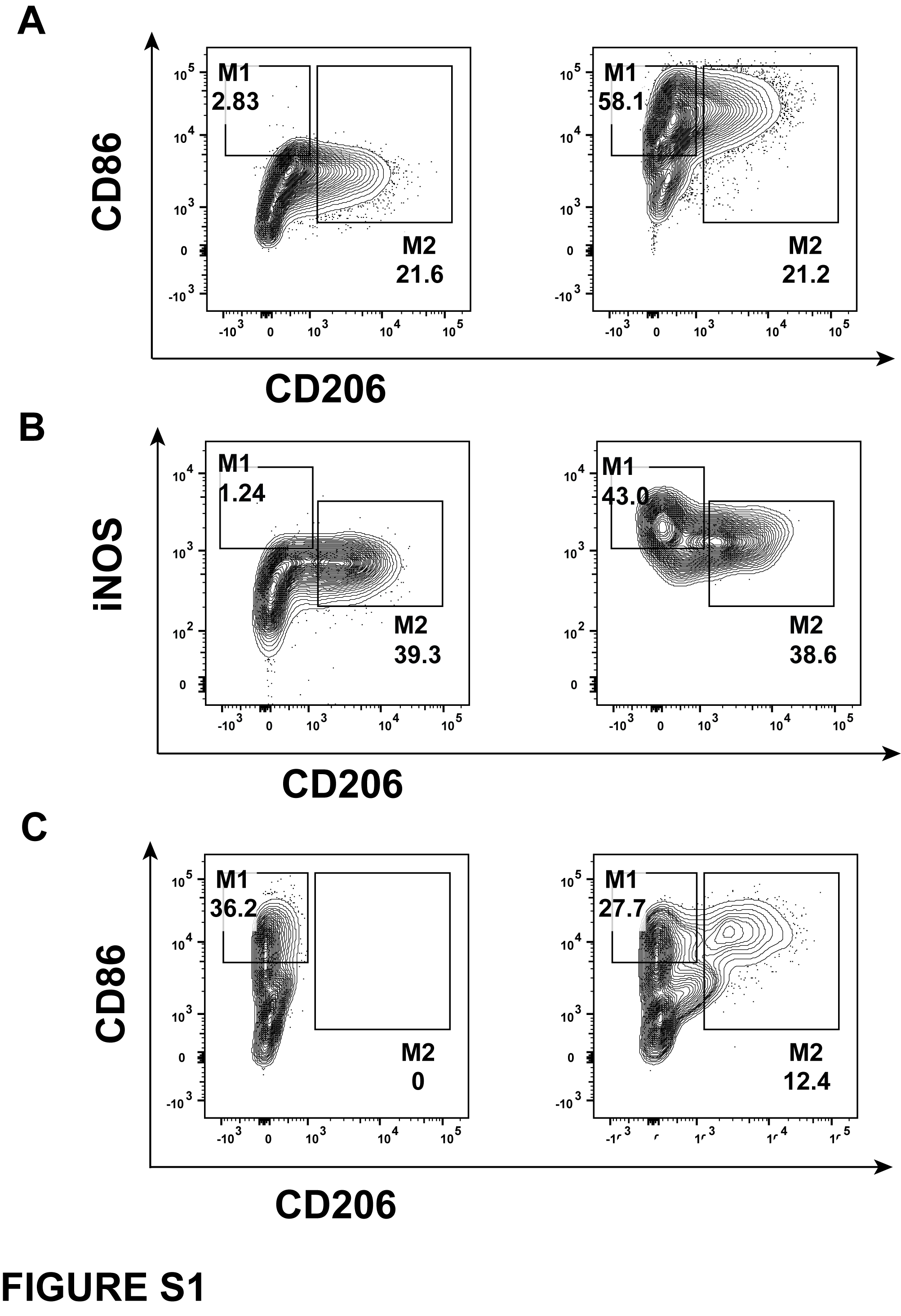

Supplement: Supplementary Figure 1 — FMO Controls of CD86, iNOS and CD206 in Flow Cytometric Analysis of Macrophages. (A) FMO Controls of CD86; (B) FMO Controls of iNOS; (C) FMO Controls of CD206. [file Image_1.tif]
